# Acetabular Fractures in the Elderly: Midterm Outcomes of Column Stabilisation and Primary Arthroplasty

**DOI:** 10.1155/2017/4651518

**Published:** 2017-01-17

**Authors:** A. Ortega-Briones, S. Smith, M. Rickman

**Affiliations:** ^1^St George's Hospital, Blackshaw Road, London SW17 0QT, UK; ^2^University of Adelaide & Royal Adelaide Hospital, Level 4 Bice Building, North Terrace, Adelaide, SA 5000, Australia

## Abstract

*Background*. Interest in arthroplasty techniques for periarticular or intra-articular fractures in the elderly/osteoporotic patient continues to rise, including for geriatric acetabular fractures. In line with this, many acetabular fracture surgeons are now undertaking acute total hip arthroplasty in elderly/osteoporotic patients. Little is known however of the outcomes of this procedure, beyond the first year after surgery.* Questions/Purposes*. We determined the clinical outcomes of a series of elderly osteoporotic patients (mean age at surgery 77.4 years) treated for acetabular fractures with column fixation and simultaneous total hip arthroplasty, at a mean of 49 months after surgery.* Methods*. 24 patients (25 hips) were reviewed at a mean of 49 months after surgery. The surgical technique employed has previously been described. Radiographs were obtained, and clinical outcomes were assessed using Harris Hip Scores and the Merle d'Aubigné score.* Results*. 14 hips were available for assessment (9 deceased, 2 lost to follow-up). No patient suffered any complications beyond the perioperative period, no acetabular components were loose clinically or on latest radiographs, and the mean Harris Hip Score was 92. All but one patient scored good or excellent on the Merle d'Aubigné score.* Conclusions*. Column fixation and simultaneous total hip arthroplasty are a viable option for complex geriatric acetabular fractures, with encouraging midterm results. We conclude that THR is a viable long-term solution in this situation provided that the acetabular columns are stabilised prior to implantation, but more research is needed to aid in overall management decision making.

## 1. Introduction

As the population of developed countries ages, new challenges are facing orthopaedic surgeons, and this ageing trend is predicted to continue [1]. As a result new techniques have been popularised for managing osteoporotic fractures, including the use of new devices such as locking plate technology, as well as augmenting fixation with substances such as bone cement [2–4] or impaction bone grafting [5]. Periarticular fractures in the elderly are becoming more common also, and with advances in arthroplasty techniques the role of acute joint replacement for periarticular fractures is expanding—particularly around the shoulder, elbow, hip, and knee. The ideal extent of this role however has yet to be defined for most joints. Patients with osteoporotic acetabular fractures represent a significant and increasingly common challenge to the orthopaedic surgeon [6]; management options are several, with no agreed algorithm defined so far to guide the treating surgeon reliably to the best option [7]. We have previously reported on the use of column fixation with simultaneous total hip replacement (THR) to allow immediate full weight bearing, but reported only early outcomes and 1-year mortality rates [8]. We now present the later clinical outcomes of the same group of patients, with follow-up to a mean of 49 months (range 33 to 69).

## 2. Patients and Methods

**Figure 1:**
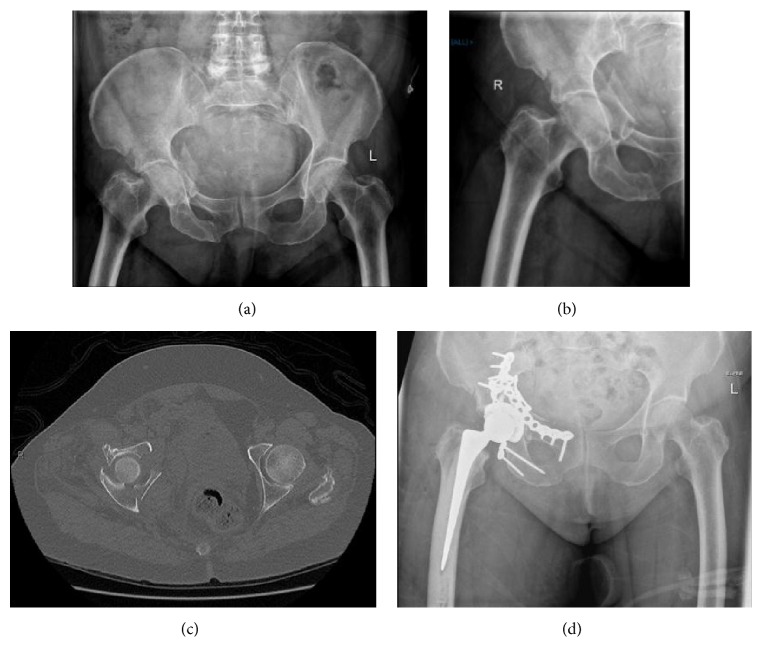
Pre- and post-op images of a typical case involving fractures of both columns. (a, b) Initial AP pelvis and film of right hip. (c) Axial CT scan of the fracture showing displacement of both columns. (d) Postoperative X-ray. Both columns have been reduced and plated, and a hip replacement was performed.

**Figure 2:**
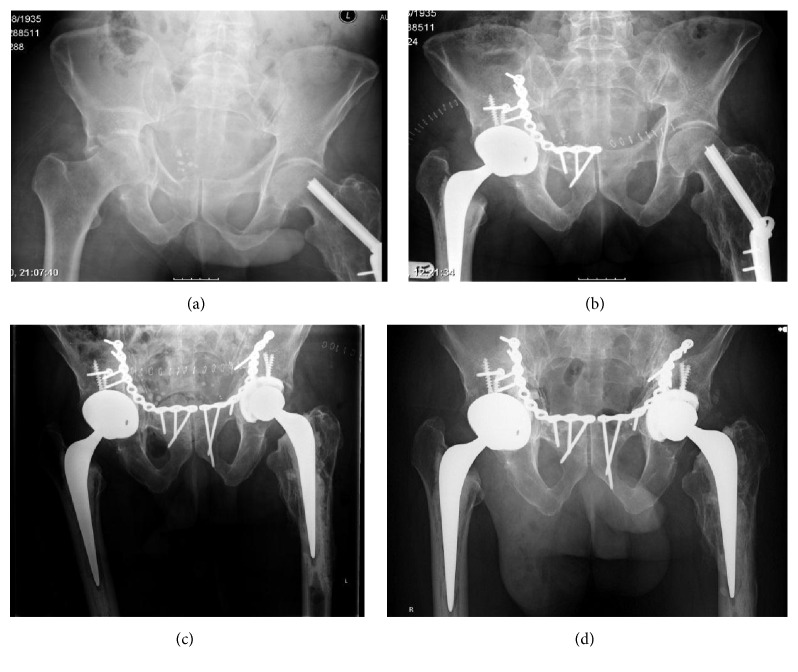
Images of patients 4 and 25, who sustained bilateral fractures at different time points. (a) Initial fracture in January 2010. (b) Postoperative view in January 2010. (c) Postoperative X-ray after second surgery in January 2012. (d) Most recent X-ray in June 2015.

The senior author's preference (MR) for management of elderly/osteoporotic acetabular fractures is simultaneous internal fixation and primary total hip arthroplasty. The technique has previously been described in detail, along with decision making processes [9]. In the absence of a past history of confirmed osteoporosis, patients were assumed to have an osteoporotic fracture based on patient age, low energy of injury and fracture pattern, and the presence of specific markers such as femoral impaction fractures or marked acetabular marginal impaction. In summary, all hip replacements were done through a Kocher-Langenbeck approach, with simultaneous fixation of the posterior column if necessary; prior to this, if there was an anterior column fracture then this was approached separately (most commonly through a modified Stoppa approach) and also secured with a plate and screws (Figure 1). This results in a stable construct, allowing a standard total hip arthroplasty to be performed using primary implants, with enough stability to allow immediate full weight bearing. All of this is done under a single anaesthetic. 25 hips in 24 patients are included in this report—the early results of which have previously been described [8]. In addition to the previously published series, one of the original 24 patients has since sustained a similar injury on the opposite side, and this new injury is also included in this paper (Figure 2).

In line with the senior authors' standard practice, none of the patients in this series were actively discharged from follow-up but were seen yearly for clinical and radiographic review—this facilitated recent reviews of most patients. Those who had not attended clinic routinely were contacted and reviewed again, although 2 patients could not be found and were lost to follow-up. In line with standard protocols, patients underwent clinical review and plain radiographs, scores were calculated for the Harris Hip Score [10] and d'Aubigné and Postel [11] score, and complications/reoperations were noted as well as current functional levels.

## 3. Results

The study group contained 25 hips in 24 patients. Nine patients had died since surgery and 2 were lost to follow-up, leaving a cohort of 14 hips in 13 patients. The mean time to follow-up of the surviving patients was 49 months, range 33 to 69. The patient details and outcomes are shown in Table 1.

The mean overall age at surgery for the group was 77.4 years (range 62 to 92). The age at surgery of patients now deceased was 78.8 (range 63 to 90) compared to 76.9 (range 64 to 92) for those still surviving. Similarly there was no detectable difference in ASA grade, comorbidities, or mechanism of injury between those patients living and deceased. No patients underwent revision surgery prior to death (or reported any problems with the operated hip), and the single reoperation which was performed for a superficial infection was described in the previous paper.

The mean Harris Hip Score was 92 (range 65 to 100), and 13 of 14 hips rated as excellent or good on the Merle d'Aubigné rating system. One patient has become wheelchair-bound secondary to general poor health and severe dementia, and thus no score was recorded for this patient. Radiologically, all fractures were healed with well-fixed acetabular components, and no cup migration was seen in any case. Radiologically no cup appeared to be at risk of loosening or revision surgery for any reason. No new complications had occurred since the perioperative period in any patient.

## 4. Discussion

This paper shows encouraging outcomes at a mean of 49 months after surgery, both in terms of implant survival and clinical results. No new complications have been seen since the perioperative period, and we therefore conclude that the use of acetabular column stabilisation and simultaneous total hip replacement with subsequent immediate full weight bearing can give excellent results, even in the longer term.

There are limitations to this paper. Firstly, the cohort is small, with only 14 surviving patients at the time of writing. Secondly, although the authors have not seen any cup migration in this series, this has only been judged on plain radiographs which may not be as accurate as using RSA technology [12]; however, we believe that no visible migration at 4 years after implantation is a positive sign, especially when combined with good functional scores. In addition, as described in our initial paper, all of the patients received trabecular metal acetabular shells. The initial stability of trabecular metal appears to be excellent, and in revision hip surgery it has been shown to osseointegrate exceedingly well [13]. However, the authors have little experience of using traditional uncemented acetabular components in this setting and cannot offer any evidence to support the use of trabecular metal over other devices; rather we have simply continued to do what appears to have been successful for us so far.

Our results are based on outcomes from 14 hips, with 9 patients having died within the study period. Using standardised mortality rates for the UK, it would be expected that approximately 5 patients would have died within this time; there is clearly an impact on mortality as a result of this injury. The presumed logic behind performing such large procedures on elderly patients comes at least in part from evidence around neck of femur fracture patients. It is now widely accepted that in that elderly population long periods of forced immobility lead to high rates of morbidity and mortality. Surgical strategies for management of neck of femur fractures in the elderly therefore almost always aim for early surgery that allows immediate full weight bearing. Evidence that the same factors affect the elderly acetabular fracture population in the same way does not exist, however tempting it may be to assume that the same applies. In addition, very few patients from the neck of femur population are deemed unfit for surgery, whereas for elderly patients with acetabular fractures it is much more likely that they will be labelled “unfit” and thus managed nonoperatively. This results in a selection bias, with published series of surgically managed elderly acetabular fractures being inherently healthier than those of neck of femur fractures. Gary et al. attempted to look at mortality with a retrospective review of cases from 3 level 1 trauma centres, cases being stratified into nonoperative, percutaneous fixation, open reduction and internal fixation, or acute total hip replacement [14]. Although they found no significant improvement of surgical over nonoperative management (and thus concluded that decisions regarding surgical management of these cases should not be based on concern over the mortality of nonoperative management) their final adjusted mortality by treatment graph shows a clinically important if not statistically significant advantage to immediate hip arthroplasty over all other forms of management. Of note, this is the only treatment modality in their groupings that would allow immediate full weight bearing.

The real difficulty remains however in deciding when to choose acute arthroplasty over the other options available for this difficult group of fractures. Risk factors for failure of more conventional methods are poorly defined within the literature but almost certainly include marginal impaction [7, 15], femoral head damage [16, 17], significant fracture comminution [12, 16], and the presence of a “gull sign” [7, 17, 18]; evidence on the use of nonoperative strategies is almost nonexistent for modern medicine, with most commonly quoted papers now being historic [19–21]. The use of acute total hip replacement for the elderly osteoporotic acetabular fracture has attractive features—it should allow immediate weight bearing and mobility, and provided that it is successful it should provide a definitive solution for the patient from a single surgical procedure. This is in contrast to internal fixation in this age group, which is almost universally followed by a period of protected weight bearing (and in this age group often immobility) and commonly leading to joint replacement surgery at a later date [7]. There have been numerous reports of the use of acute arthroplasty for acetabular fractures in the past, although posterior fracture patterns predominate, and most did not allow early weight bearing. One relevant paper by Solomon reported the early outcomes of a very similar patient group, but using a different method of oversized acetabular components; whilst also achieving some success, their RSA studies showed that 3 of their 10 cases migrated a significant amount [12]. Many commonly quoted papers are no longer truly relevant, using cemented cups, small femoral heads, and older stem designs [22, 23]. In addition, a number of papers previously published on outcomes of “elderly” patients with acetabular fractures define elderly as over 55 or 60 [7, 18, 24, 25]. Perhaps the reader can decide if this is a true definition of the word, but in orthopaedic terms the difficult population is typically patients aged over 70. All but 2 of our patients were over 70, but little attention is paid to this specific group within the literature. Not only are today's patients older, but the fractures seen in the current orthopaedic climate are often different to those seen 50 years ago, when the current classification system of Letournel was devised. Underlying bone quality is different, and activities have changed in the elderly, resulting in the generation of a different set of fracture patterns with a predominance of anterior column damage combined with often incomplete posterior fractures [26]. As such, it is not unreasonable to assume that we may need different solutions to the problem in addition to those that were popularised in the past. Duarka et al. [7] published a systematic review of the literature in 2014 on the outcome of patients over the age of 55 with acetabular fractures. Their conclusions were that there is little or no clear evidence that any one treatment strategy is better than any other and that there was little published either on the nonoperative management of this group or to delineate the outcomes between early and delayed arthroplasty.

Patients considered for surgery in this age group will fall into one of 3 broad groups: those who would not survive the perioperative period, those who will die within a year, and those who will live for a substantial period of time. In an ideal world, accurate prediction of this grouping would allow the surgeon to manage patients accordingly, and in all probabilities the first group is best managed nonoperatively and the second with internal fixation. The third group however ideally requires a single long-lasting surgical procedure—the inherent problem being the high revision rate of internal fixation in this group, especially beyond 1 year.

## 5. Conclusion

This is the first report that the authors are aware of showing midterm outcomes for elderly patients undergoing acute total hip replacement for acetabular fractures, using trabecular metal and allowing immediate full weight bearing. No cups were loose at a mean of 49 months and late complications have not been seen. We conclude that THR is a viable long-term solution in this situation provided that the acetabular columns are stabilised prior to implantation, but more research is needed to aid overall management decision making.

## Figures and Tables

**Table 1:** Details of surviving patients.

Patient	Age	Sex	Walking aids	Harris Hip Score	Merle D'A Score	Months to present follow-up
3	79	M	None	96	Excellent	57
4	75	M	1 stick	90	Good	65
5	65	M	None	100	Excellent	49
6	71	M	None	100	Excellent	50
11	70	M	None	65	Fair	51
12	73	M	None	100	Excellent	69
14	86	M	1 stick	90	Good	47
15	87	M	1 stick	84	Good	45
16	77	F	None	96	Excellent	45
17	64	M	None	100	Good	40
18	75	F	None	84	Good	50
19	72	M	None	96	Excellent	38
21	84	F	Lost	Lost	Lost	Lost to follow-up
22	92	F	Wheelchair	n/a	n/a	33
23	84	M	Lost	Lost	Lost	Lost to follow-up
25	77	M	1 stick	96	Good	41
